# Burkitt Lymphoma of Thyroid Gland in an Adolescent

**DOI:** 10.1155/2014/187467

**Published:** 2014-04-24

**Authors:** K. Cooper, A. Gangadharan, R. S. Arora, R. Shukla, B. Pizer

**Affiliations:** ^1^Alder Hey Children's Hospital, Eaton Road, Liverpool L12 2AP, UK; ^2^Max Super Speciality Hospital, Saket, New Delhi, Delhi 110017, India

## Abstract

Burkitt Lymphoma is a highly aggressive form of non-Hodgkin's lymphoma that in nonendemic areas has abdominal primary sites. We report a very rare case of Burkitt lymphoma of the thyroid gland presenting as a rapidly growing thyroid swelling in a 14-year-old white Caucasian British male with no preexisting thyroid or medical problems. The diagnosis was confirmed by an open wedge biopsy following a fine needle aspiration. The patient was treated according to the Children's Cancer and Leukaemia Group guidelines for BL—Group B protocol and currently is in remission.

## 1. Introduction

Burkitt lymphoma (BL) is a highly aggressive form of non-Hodgkin's lymphoma (NHL) characterised histopathologically by a mass of diffuse, small noncleaved B-cell lymphocytes with a “starry sky” appearance. Two forms of BL are commonly distinguished: the “endemic” or African form, first described in 1958 [1], and the “sporadic” form, found in the rest of the world including North America, Northern and Eastern Europe, and the Far East.

The head and neck are the primary sites of presentation in 50–70% of cases in endemic areas [1]. In nonendemic areas, abdominal tumours predominate and head and neck disease is seen in 8–30% of cases.

Here we describe the clinical presentation and management of a case of BL in an adolescent boy that occurred in a rare site.

## 2. Case Presentation

A 14-year-old white British Caucasian boy presented with a 3-month history of malaise, lethargy, and weight loss. There was no history of fever or any other thyroid related symptoms. Past and family history were noncontributory and there was no recent foreign travel. On examination he was found to have a large predominantly left-sided firm thyroid swelling. Clinical examination was otherwise unremarkable with no lymphadenopathy or hepatosplenomegaly or signs of hypo/hyperthyroidism. Baseline haematological and biochemical investigations on admission were within normal limits (Hb 136 g/L, WCC 9.1 10^9^/L, CRP <4 mg/dL). Levels of thyroxine, TSH, and calcitonin were normal. Lactase dehydrogenase (LDH) was slightly elevated at 352 U/L. Thyroperoxidase was elevated (1277 IU/mL) and thyroid stimulating hormone receptor antibodies (TRAb) of <5 U/L. CSF analysis showed no tumour cells. EBV PCR was negative as was HIV-I and HIV-II.

**Figure 1:**
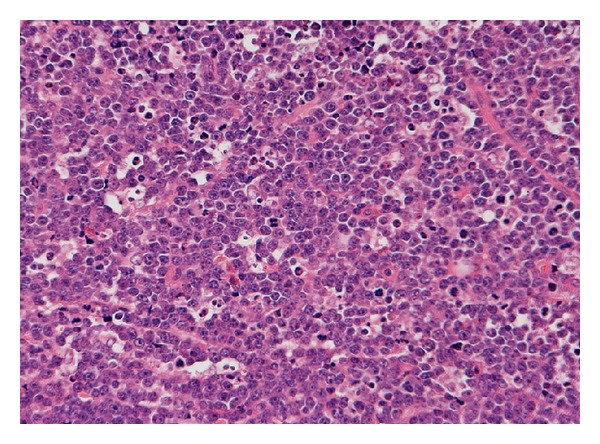
Histology from open wedge biopsy. The tumour showed diffuse sheets of lymphoid cells punctuated by tingible body macrophages (H&E 10X).

A fine needle aspiration biopsy was carried out and was suspicious of NHL. The diagnosis of Burkitt type NHL (BL) was made following an open wedge biopsy. On microscopic examination, the tumour was composed of diffuse sheets of lymphoid cells (Figure 1) having round nuclei with coarse chromatin and conspicuous single to multiple nucleoli and scanty amphophilic cytoplasm. Numerous mitotic figures, apoptosis, and scattered tingible body type macrophages were also present. Tumour cells were positive for CD79A, CD20, and CD10 and negative for TdT and T cell markers (CD3, CD4, and CD8). Ki 67 showed proliferation index approaching 100%. Immunohistochemistry for EBV was negative. The diagnosis of BL was confirmed on fluorescence in situ hybridisation that showed tumour cell positivity for the t(8; 14) translocation.

**Figure 2:**
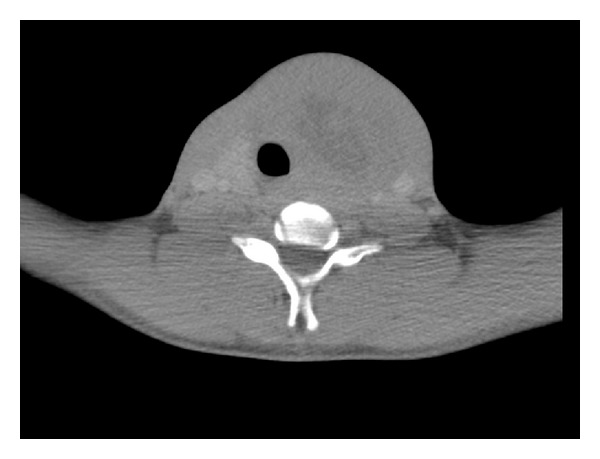
Staging CT showing a large (6.7 cm × 5.8 cm × 6.1 cm) heterogeneous mass in the left lobe and isthmus of the thyroid.

Staging CT (Figure 2) showed a large (6.7 cm × 5.8 cm × 6.1 cm) heterogeneous mass in the left lobe and isthmus of the thyroid which extended craniocaudally from level of C4/5 to T1/2, with some evidence of intratumoural necrosis. There was significant mass effect with displacement and compression of the trachea along with compression of the left carotid artery and lateral displacement of the left jugular vein. There were two small indeterminate lung nodules. Abdominally there were two focal cortical abnormalities in the right kidney and an ill-defined attenuation in the pancreatic head. There was no evidence of bone marrow involvement.

He was treated according to the Children's Cancer and Leukaemia Group guidelines for BL—Group B protocol. This consisted of initial induction chemotherapy with COP (cyclophosphamide [300 mg/m^2^], vincristine [1 mg/m^2^], and prednisolone [60 mg/m^2^—7 days]) followed by 2 courses of COPADM (doxorubicin 60 mg/m^2^, methotrexate 3 g/m^2^, cyclophosphamide 500 mg/m^2^/day—5 days, vincristine [2 mg/m^2^], and prednisolone [60 mg/m^2^—5 days]) and two courses of CYM (cytarabine 100 mg/m^2^/day—5 days and methotrexate 3 g/m^2^) chemotherapy. This was accompanied by intrathecal chemotherapy (the CSF contained no tumour cells at diagnosis). The patient had a good response to treatment with a rapid decrease in the size of the thyroid mass and resolution of the lung, kidney, and pancreas metastases. Reassessment after the first course of CYM showed a residual thyroid mass of 15 mm × 30 mm but with resolution of the lung, kidney, and pancreas metastases.

Because of residual radiological abnormality, and in accordance with the CCLG NHL protocol, a hemithyroidectomy was performed with the residual lesion showing no evidence of tumour cells on histological examination. Chemotherapy was then completed and he is now disease-free 3 years after end of treatment.

## 3. Discussion

Cancer of the thyroid gland is rare in childhood, forming about 0.4% of all paediatric malignancies in Great Britain [2]. The most common types of thyroid malignancies include papillary carcinoma (68%), follicular carcinoma (11%), and medullary carcinoma (17%) [2]. Primary lymphoma of the thyroid (non-Hodgkin's lymphoma) is even rarer accounting for 2–3% of paediatric thyroid tumours [2, 3].

Large adult population-based [4] as well as retrospective clinic-pathological case series [5] suggest that primary thyroid NHL typically occur in middle- to older-aged persons in the setting of preceding lymphocytic thyroiditis and have a predilection for females. The common histological subtypes are diffuse large B-cell lymphoma or mucosa-associated lymphoid tissue (MALT) lymphoma or a mixture of both [4–6]. Primary BL of the thyroid is very rare with a few isolated case reports in adult patients [7–10]. There are no published case-series of primary thyroid NHL in children and an extensive literature search only identified one previous case-report of primary BL of the thyroid [11]. Involvement of thyroid gland secondary to abdominal BL has been more widely reported in the paediatric literature [12–17].

The one previous paediatric case was also in an adolescent boy but who presented more acutely with a 1-day history of an enlarging lower neck mass and dyspnea [11]. He had normal thyroid function tests and no metastatic spread. There are four previously reported cases in adults to date [9, 10, 18, 19]. Two of these adult cases presented due to the thyroid mass causing tracheal compression and thus airway compromise. In all of these patients chemotherapy rapidly shrunk the mass with one of the patients having complete resolution within 36 hours. All of the patients underwent fine needle aspiration, incisional thyroid, and bone marrow biopsies. Hashimoto's thyroiditis is present in 85% of primary lymphoma of the thyroid in adults [1]. The hypothesis is that chronic antigenic stimulation secondary to the autoimmune disorder leads to chronic proliferation of lymphoid tissue, which eventually undergoes a mutation that leads to the development of lymphoma.

Extranodal low-grade B-cell lymphoma of mucosa-associated lymphoid tissue (MALT) type occurs in the gastrointestinal tract, salivary gland, thyroid, orbit, lung, and breast. EBV, malaria, HIV and AIDS, radiation exposure in any form, and plant chemicals (4-deoxyphorbol ester) which reduces EBV-specific cytotoxic T-cell function have been implicated in aetiology of BL. Even though there are different types of BL, endemic and sporadic, all tumours contain the same chromosomal translocations, which culminate in the deregulation of the oncogene c-myc. The translocations involve the myc location (8q24) and one of the immunoglobulin loci on chromosomes 2, 14, or 22 [20]. The reciprocal translocation t(8 : 14) occurs in approximately 80% of tumours [21].

MALT lymphomas and diffuse large B-cell lymphoma account for nearly all thyroid lymphomas, with MALT type having a better prognosis. The majority of MALT lymphomas are of B cell origin and are thought to arise due to antigenic stimulation from chronic infection and inflammation. In the thyroid gland they are often associated with autoimmunity, for example, in Hashimotos thyroiditis.

Diffuse large B-cell lymphoma is the most common type of thyroid lymphoma of B cell origin accounting for 70% of cases. Around 40% appear to originate as MALT lymphoma. It is a highly aggressive subtype with almost 60% presenting as disseminated disease and with a 5-year prognosis of less than 50%.

To the authors knowledge this is only the second reported case of BL of the thyroid gland in children and adolescence. Although rare a diagnosis of BL should be added to the differential diagnosis of thyroid masses in the paediatric population.
